# End-stage ADPKD with a low-frequency PKD1 mosaic variant accelerated by chemoradiotherapy

**DOI:** 10.1038/s41439-024-00273-0

**Published:** 2024-03-28

**Authors:** Hiroaki Hanafusa, Hiroshi Yamaguchi, Naoya Morisada, Ming Juan YE, Riki Matsumoto, Hiroaki Nagase, Kandai Nozu

**Affiliations:** 1 Department of Pediatrics, Kobe University Graduate School of Medicine, Hyogo, Japan; 2 Department of Genetics, Hyogo Prefectural Kobe Children’s Hospital, Hyogo, Japan; 3 Department of Neurology, Kobe University Graduate School of Medicine, Hyogo, Japan

**Keywords:** Genetics research, End-stage renal disease

## Abstract

Autosomal dominant polycystic kidney disease (ADPKD) is commonly caused by *PKD1*, and mosaic *PKD1* variants result in milder phenotypes. We present the case of a 32 year-old male with chronic active Epstein–Barr virus who underwent bone marrow transplantation with chemoradiotherapy at age 9. Despite a low-frequency mosaic splicing *PKD1* variant, he developed severe renal cysts and end-stage renal disease in his 30 s. This case highlights how environmental factors may contribute to the genetic predisposition to ADPKD.

Cystic kidneys frequently lead to end-stage renal disease (ESRD). Autosomal dominant polycystic kidney disease (ADPKD) is the most common form of PKD^1^. Genetic variants of *PKD1* or *PKD2*, which encode polycystins (PCs) 1 and 2, respectively, are the most common variants associated with ADPKD^1^. Mosaic variants of *PKD1* are also involved in cases of PKD, and these patients reportedly exhibit milder phenotypes^2^.

Chronic active Epstein–Barr virus (EBV) disease (CAEBV) is a lethal syndrome caused by persistent EBV infection. Current treatments for CAEBV include chemoradiotherapy (CRT) and hematopoietic stem cell transplantation^3^. CRT during childhood can cause various future complications, such as secondary cancers, especially in tissues with slow turnover, such as the brain and kidneys^4^.

Herein, we report the case of a 32 year-old male who was referred to our department for genetic testing. The patient was born to nonconsanguineous parents via vaginal delivery without complications at 39 weeks of gestation. The patient had no significant family history. The patient was healthy until he was diagnosed with CAEBV at 5 years of age. The patient was treated with interferon, interleukin, and high-dose steroid therapy for 2 years. When the patient was 9 years old, he underwent total body radiation (12 Gy), high-dose chemotherapy/myeloablative conditioning and bone marrow transplantation (BMT) with his father as a donor. Clusters of clonic seizures occurred 25 days after BMT, and the patient was diagnosed with epilepsy. Subsequently, he developed refractory epilepsy and was prescribed multiple antiseizure medications, and a vagus nerve stimulator was implanted. He also had significant medical problems, such as chronic kidney disease (blood test results revealed a blood urea nitrogen level of 36.6 mg/dL, creatinine level of 2.66 mg/dL, and estimated glomerular filtration rate of 26.3%), high blood pressure, hypothyroidism, retinochoroidal atrophy, bilateral cataracts, and parkinsonism. Recent brain magnetic resonance imaging (MRI) analysis revealed hyperintensity in both basal ganglia on T1-weighted imaging (T1WI) (Fig. 1a). Additionally, a ring-shaped angioma with hyperintensity on T1WI (Fig. 1b) and hypointensity on T2WI was observed in the left frontal lobe (Fig. 1c). Computed tomography (CT) of the head revealed calcifications in the bilateral basal ganglia and cerebral lesions (Fig. 1d). Abdominal CT revealed severe bilateral PKD (Fig. 1e). The patient is now waiting for a kidney transplant while undergoing peritoneal dialysis.

**Fig. 1:**
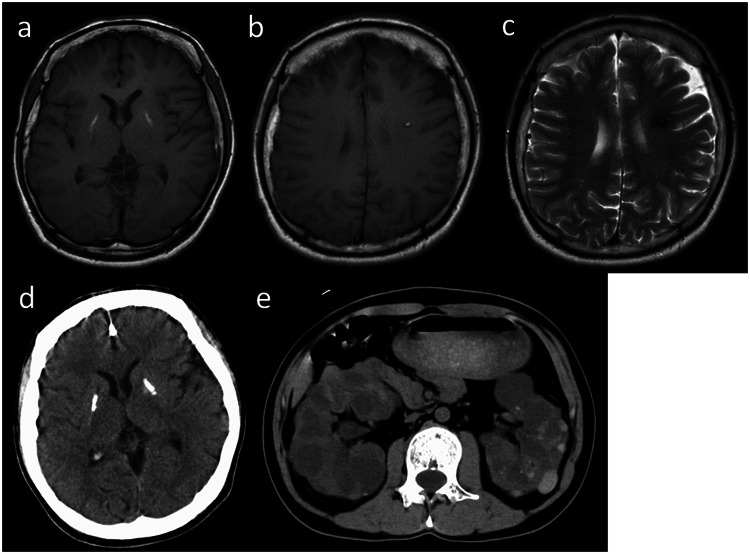
Imaging results of the patient. Brain magnetic resonance imaging (MRI) and head computed tomography (CT) were performed when the patient was 32 years old. **a**, **b** T1 weighted imaging (T1WI), **c** T2WI, **d** head CT, and **e** abdominal MRI.

The patient and his parents requested genetic testing and provided informed consent. A gene panel test and whole-exome sequencing (WES) were performed. Customized panel analysis revealed a novel mosaic splice site variant of *PKD1* [NC_000016.10 (NM_001009944.3):c.3295+2 T > A] in genomic DNA derived from both hair and urine samples. Detailed information about the analysis and pathological evaluation of each variant is described in the Supplementary Methods. The identified variant is not registered in population databases, such as gnomAD and jMorp, and was evaluated as likely pathogenic (PVS1 + PM2) according to the ACMG–AMP guidelines. The variant allele frequencies in genomic DNA derived from hair and urine samples were 16.5% (17/107) and 10.6% (11/104), respectively (Fig. 2a). Customized panel analysis of genomic DNA from urine-derived cells did not reveal other pathogenic or likely pathogenic variants with allele frequencies >10%, indicating that no second hit of the other *PKD1* allele occurred. Direct sequencing analysis revealed the same variants that were detected using next-generation sequencing of genomic DNA from hair- and urine-derived cell samples, and the peaks of the variants were lower than those of the wild-type alleles (Fig. 2b). WES analysis revealed no pathogenic germline variants in other genes.

**Fig. 2:**
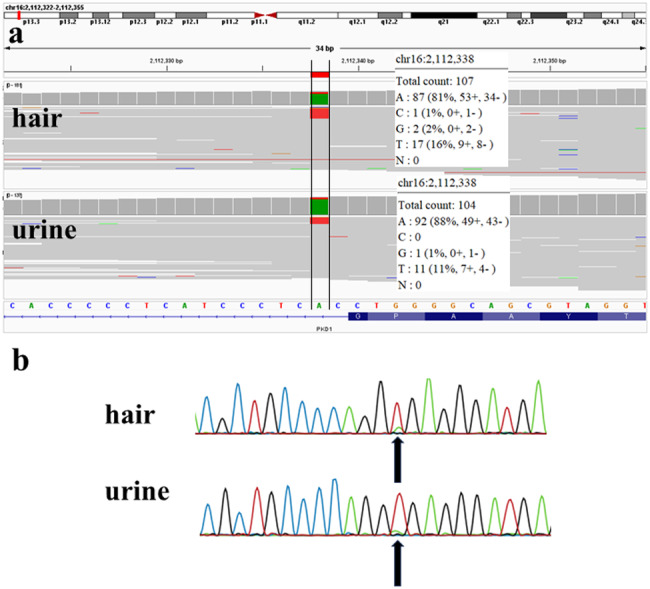
Results of customized panel analysis of genomic DNA and direct sequencing of genomic DNA from hair and urine samples. **a** The allele frequency of the variant [NC_000016.10(NM_001009944.3):c.3295+2 T > A] was 16.5% (17/107) for genomic DNA from hair samples and 10.6% (11/104) for genomic DNA from urine samples. **b** The variant [NC_000016.10(NM_001009944.3):c.3295+2 T > A] was identified in genomic DNA from hair and urine samples. The peaks of the variant are low, suggesting somatic mosaicism.

Despite a low variant allele frequency of 10.6% in urine-derived cells, the patient developed severe bilateral PKD in his early 30 s and needed dialysis and renal transplantation. Hopp et al. noted that patients with *PKD1* mosaic variants exhibited milder symptoms^2^. In this patient, the variant allele frequency was low (~10%); however, the renal symptoms were severe at 33 years of age. The reported median renal survival of individuals with end-stage kidney disease carrying *PKD1* truncating variants was 55.6 years of age^5^.

The loss of function of the receptor/channel complex formed by the proteins encoded by *PKD* genes below an undetermined threshold triggers cyst development, which leads to the dysregulation of multiple metabolic processes and cyst growth^6^. Disease severity is determined by the effect of the primary variant on the activity of PC1 and PC2, in addition to the presence of other variants and environmental factors such as renal injury^7^. In the present case, the allele frequency of the *PKD1* variant was ~10%; therefore, it did not strongly decrease PC1 activity, and no other variants in inherited renal disease genes, including *PKD1*, were found in urine-derived genomic DNA.

Our case suggests that, even in a patient with *PKD1* with a very low-frequency mosaic variant, CRT may exacerbate PKD. Radiotherapy causes DNA breaks and subsequent cell death, and an increasing number of patients who undergo chemotherapy experience side effects^4^. Chemotherapy also increases the risk of late effects^8^.

CRT for CAEBV is potentially associated with late adverse effects. In the present case, the patient developed several symptoms in addition to severe PKD and ESRD. He presented with a cavernous hemangioma and calcifications in the brain and developed epilepsy and parkinsonism in his early 30 s. Although co-occurrence of cerebral cavernous malformations and ADPKD is often observed, cerebral cavernous malformations can also occur after cerebral radiation^9^. Refractory epilepsy has also been reported as a late effect of chemoradiation^10^. There are also reports of intracranial calcification in the basal ganglia and parkinsonism caused by radiation therapy^11^. Furthermore, hypothyroidism and retinochoroidal atrophy have been reported as late adverse effects of CRT^12^.

This case emphasizes the potential influence of environmental factors on genetic predisposition in ADPKD patients. Furthermore, it highlights that late adverse effects of childhood CRT are a serious clinical concern, particularly in patients with a genetic predisposition for PKD, which necessitates long-term follow-up in these patients.

## Supplementary information

Supplementary Methods

## Data Availability

The relevant data from this Data Report are hosted at the Human Genome Variation Database at 10.6084/m9.figshare.hgv.3397.
