# Supplementary material for: End-stage ADPKD with a low-frequency PKD1 mosaic variant accelerated by chemoradiotherapy
Source: Hum Genome Var. 2024 Mar 28;11:17. doi: 10.1038/s41439-024-00273-0 (PMC10978896; doi:10.1038/s41439-024-00273-0)
Supplement: Supplementary file 1 — Supplementary Methods [file 41439_2024_273_MOESM1_ESM.docx]

**Supplementary Methods**

**Genetic analysis**

Because the patient had undergone bone marrow transplant when he was 9 years old, genomic DNA was extracted from urine-derived cells using the DNeasy Blood & Tissue kit (QIAGEN, GmbH, Hilden, Germany). Next-generation sequencing (NGS) was performed using an original custom gene panel for inherited renal diseases. This panel contained 186 genes, including *PKD1*, and the complete list of genes included in the panel is presented in Supplementary Table S1. NGS samples were prepared with SureSelect PostPool Custom Tier2 (0.5–2.9 Mb) and sequenced using MiSeq platform (Agilent Technologies, Santa Clara, CA, USA). SureCall software (v4.2.1.10; Agilent Technologies) was used for data analysis. Subsequently, to confirm the mosaicism rate in urine-derived cells, DNA was extracted from urine using a Quick-DNA Urine Kit (Zymo Research, Orange, CA, USA) and analyzed using the same method. *PKD1* variants detected by NGS were also analyzed via direct sequencing of genomic DNA from hair and urine samples. To exclude other genetic diseases, whole-exome sequencing was performed using NovaSeq 6000 (Illumina, San Diego, CA, USA) and SureSelect Human All Exon V6 (Agilent Technologies). Reads were mapped to human genome assembly hg38 the Burrows-Wheeler Alignment-Maximal Exact Match algorithm in Burrows-Wheeler Aligner software (v0.1.17).^1^ To obtain BAM files, PCR duplicates were marked with Picard MarkDuplicate v2.18.29 (<http://broadinstitute.github.io/picard>.), and the base quality score was recalibrated using the Genome Analysis Toolkit (GATK; v4.3.0.0). Variant calling was then performed using the GATK Haplotype caller, and VCF files were obtained and annotated using ANNOVAR (v2019Oct24).^2^

**References**

1. Li H, Durbin R. Fast and accurate short read alignment with Burrows-Wheeler transform. *Bioinformatics* 2009; **25**: 1754–1760.

2. Wang K, Li M. Hakonarson H. ANNOVAR: functional annotation of genetic variants from high-throughput sequencing data. *Nucleic Acids Res* 2010; **38**: e164.
